# Life Disruptions, Symptoms Suggestive of Endometriosis, and Anticipated Stigma Among College Students in the United States

**DOI:** 10.1089/whr.2021.0072

**Published:** 2021-12-27

**Authors:** Jhumka Gupta, Lauren Cardoso, Samantha Kanselaar, Anna M. Scolese, Alzahra Hamidaddin, Anna Z. Pollack, Valerie A. Earnshaw

**Affiliations:** ^1^ Department of Global and Community Health, College of Health and Human Services, George Mason University, Fairfax, Virginia, USA.; ^2^ School of Social Policy and Practice, University of Pennsylvania, Philadelphia, Pennsylvania, USA.; ^3^ Department of Human Development and Family Sciences, College of Education and Human Development, University of Delaware, Newark, Delaware, USA.

**Keywords:** endometriosis, pain, stigma, women's health, college health

## Abstract

***Background:*** Endometriosis is a chronic gynecological condition impacting 1 in 10 women of reproductive age. Research with adult women documents high levels of disruptions in academic, professional, family, and social lives due to pain. Less research has been conducted with college-aged populations. Although stigma has been noted as a key factor in contributing to diagnostic delay, little research has focused on examining stigma, endometriosis symptoms, and disruptions. This study aims at documenting the frequency of social, academic, and work disruptions experienced by college-aged women due to symptoms suggestive of endometriosis and at examining the relationship between disruptions and anticipated stigma.

***Methods:*** An online survey was conducted in April 2019 among a nationally drawn sample (*N* = 468) of undergraduate women to assess symptoms suggestive of endometriosis, disruptions to daily life, and stigma experiences.

***Results:*** High levels of life disruptions due to symptoms suggestive of endometriosis were documented (88% any disruption, 82.7% social, 58.8% academic, and 34.4% work). Adjusted analysis (accounting for demographics and symptoms) showed that any disruptions, social disruptions, academic disruptions, or work disruptions were significantly associated with a higher mean anticipated stigma score (*β* = 0.37, 95% confidence interval [CI]: 0.15–0.59; *β* = 0.32, 95% CI: 0.13–0.51; *β* = 0.32, 95% CI: 0.17–0.46; *β* = 0.55, 95% CI: 0.23–0.54; respectively).

***Conclusions:*** Many young women experience disruptions that affect their academic, work, and social lives. These disruptions due to symptoms suggestive of endometriosis also impact stigma experiences. More interventions tailored to address stigma and minimize disruptions are needed in public health.

## Introduction

Endometriosis is a chronic condition that is estimated to impact up to 10% of women and girls of reproductive age worldwide,^1,2^ and up to 30%–50% of symptomatic women.^3^ Women with endometriosis often experience debilitating symptoms, including painful and irregular menstruation, back and pelvic pain, bowel and urinary disorders, and infertility.^4–8^ The condition impacts women's physical functioning, including mobility restrictions, as well as emotional well-being.^9–11^

Disruptions to social, educational, and professional obligations have also been reported.^12^ Although much of the research on disruptions has been qualitative, one quantitative study estimated that women with endometriosis miss an average of 19.3 work days annually.^13^ Moreover, existing research regarding the socioeconomic impacts of endometriosis focuses on adult women. Emerging qualitative data indicate that adolescents and young adult women also experience disruptions.^14^

As young adult women face challenges related to new social roles and responsibilities (*e.g.*, managing their own health, making future decisions, navigating new independence from parents), disruptions in social, academic, and work spheres due to pain and other symptoms common with endometriosis can make navigating these paths even more difficult. These disruptions may increase young adult women's vulnerability to stigma. People living with chronic illness can internalize, experience, and anticipate stigma—a social process that results in the devaluation and discrediting of people with various characteristics, attributes, and/or identities.^15,16^

According to stigma theorists, chronic illnesses are stigmatized both because they are assumed to render individuals as poor social exchange partners (*i.e.*, individuals with chronic illnesses may be perceived to need more than they can give in interpersonal relationships) and may be mistaken as infectious and therefore a threat to others.^16,17^ Research has shown that bowel dysfunction, painful intercourse, and chronic pain, symptoms that are commonly experienced by women and girls with endometriosis, can also be stigmatized by others.^14^

In addition, research from the United States and other global regions has underscored that menstruation itself is socially stigmatized, in part because it is a marker of femaleness.^13^ Thus, symptoms common in endometriosis and associated with menstruation (*e.g.*, debilitating pelvic pain with menses) are stigmatized.

Young adult women experiencing disruptions associated with endometriosis may have elevated concerns surrounding anticipated stigma. Endometriosis is a concealable stigmatized status; that is, it is not immediately observable by or visible to others.^18^ People with concealable stigmatized statuses face threat of discovery (*i.e.*, others becoming aware of their stigmatized status within situations that may make their status more relevant or visible to others).^18^ Young adult women experiencing disruptions due to endometriosis symptoms may be under increased threat of discovery when others attempt to understand why they may have withdrawn from a social activity, were absent from class, or missed work.

This threat of discovery, in turn, may lead women to anticipate stigma, or expect discrimination, prejudice, and/or stereotyping from others in the future.^19^ Evidence suggests that endometriosis symptoms are often trivialized by others (*e.g.*, family members, friends, health care workers), and women and girls conceal endometriosis symptoms due to fear of ostracization and trivialization from others.^20–23^

Anticipated stigma undermines the mental and physical well-being of people living with a wide range of chronic illnesses. Anticipated stigma is a stressor, and people with chronic illnesses who anticipate more stigma from work colleagues as well as friends and family report experiencing greater stress.^24^ In addition, people with chronic illnesses who anticipate more stigma from friends and family report less social support, perhaps as a result of withdrawing or isolating from others from whom they expect negative reactions.^24^ Evidence further suggests that people with chronic illnesses who anticipate stigma from health care providers access care less frequently than what may be medically recommended and those who anticipate stigma from community members are less adherent to medical treatments.^25,26^

Anticipated stigma is additionally associated with worse mental (*e.g.*, depressive and anxiety symptoms) and physical (*e.g.*, illness symptoms, comorbidities) health.^19,27,28^ Among adult women with endometriosis, anticipated stigma may undermine help-seeking and promote a tendency to live with the condition in private.^21,29^ Importantly, emerging adulthood may be a sensitive period for experiences of stigma, or a developmental stage when stigma may have an especially pronounced effect on well-being.^30,31^

Young adult women may anticipate stigma more frequently than older adult women, as they transition between social, educational, and professional settings.^32^ They may also be more harmed by anticipated stigma as they are still developing coping and other resilience resources.^33,34^

A recent review concluded that endometriosis stigma is understudied and called for greater research to understand and intervene in stigma to improve the well-being of women experiencing endometriosis.^35^ The current study aims at addressing this gap by: (1) documenting the frequency of disruptions (social, academic, and work) due to symptoms suggestive of endometriosis, and (2) examining the relationship between disruptions due to symptoms and anticipated stigma among a sample of young women enrolled in undergraduate studies.

## Methods

### Overview

An online survey was conducted in April 2019 among a nationally drawn sample of undergraduate women ages 18–25 to assess symptoms suggestive of endometriosis, disruptions to daily life, and stigma experiences.

### Survey development

The survey was initially developed utilizing the research team's earlier study^14^ on perceptions of endometriosis, as well as additional research on menstrual health and its associated life disruptions, endometriosis and its socioeconomic impact, chronic health issues that disrupt daily life, and stigma experiences and beliefs of people living with chronic health conditions.^12,19,36,37^ Survey questions from these studies were adapted for the current study; original questions were also included. After the development of the survey tool, cognitive interviews were conducted with 10 participants who met the inclusion criteria.

Participants took the survey with a research assistant present. The research assistant asked probing questions for certain survey items to assess the participant's understanding of terms, thought process in selecting an answer, and any challenges or confusion related to the survey questions. For example, participants were asked, “Did you experience any confusion or uncertainty when trying to answer this question? If so, please explain what was confusing.” All cognitive interviews were audio recorded. The results were reviewed by the research team, and several survey items were revised.

After the cognitive interviews, the survey was piloted with 50 participants. Participants were contacted by our data collection partner, Qualtrics, a research firm specializing in online data collection. Data collection procedures are detailed next. After the pilot, more revisions were made—specifically some modules were removed and others reorganized. In addition, some quality checks were added to ensure data were of high quality. For example, participants were asked before starting the survey, “Do you commit to thoughtfully provide your best answers to the questions in this survey?” Only those who selected “I will provide my best answers” (vs. “I will not provide my best answers” or “I can't promise either way”) were able to begin the survey.

### Data collection

A convenience sample of undergraduate women was selected with the help of Qualtrics. Potential respondents were contacted via an email that introduced the study and described study procedures. If interested, potential participants were directed to the online informed consent. This described in detail the purpose of the study, the procedures, compensation for completing the survey ($15), risks and benefits, and the voluntary nature of participation. Potential participants provided consent by completing the survey. The 30-minute survey covered topics related to menstrual and other health issues, menstrual health-related disruptions to daily life, and stigma. The study received Institutional Review Board approval from George Mason University.

### Participants & quality checks

Potential participants answered several screener questions to assess eligibility. Inclusion criteria included: aged 18–24, female sex at birth, and enrolled as a university student. The study oversampled for racial minorities. Initially, 2715 participants showed interest; 1460 were eligible; and 515 fully completed the survey.

The research team created a rubric of “red flags” for potentially poor-quality responses. These red flags included: completing the survey in less than 11 minutes, reporting numeric responses for multiple questions that did not align (*e.g.*, diagnosed with endometriosis many years before menstrual period started), and straight-lined multi-item scales. Those who had five or more red flags were considered “poor quality” and were removed. A total of 14 respondents were removed, leaving 501 participants. Another measure of quality control was through the verification of IP address and zip codes provided by participants.

This was done by reviewing every other block of 10 participants' zip codes and IP address (*n* = 311). The IP addresses were matched to zip codes by using a website that geolocates the IP address to zip code and were then checked to see whether they matched their respective zip codes or fell within 50 miles of their respective zip codes. In total, 99.95% of respondents IP addresses matched their zip codes; 15 respondent's zip codes and IP addresses did not match, and 40 were missing zip code information.

For this study's analytic sample, 468 respondents were included. Respondents who reported that they had never had a period, had not had a period in the past year, or had not had any of the 26 endometriosis-related symptoms were omitted.

### Measures

#### Demographics

Demographic variables were adapted from previous health research with university students^38^ and included age (response options from 18 to 25), sex assigned at birth (male or female), enrollment status (full- or part-time), living on campus, relationship status (not in a relationship, in a relationship but not cohabitating, in a relationship and cohabitating), and self-reported health (excellent, very good, good, fair, poor, do not know). Respondents were asked to self-report their race, and this race variable was collapsed to four categories (White, Black, Hispanic or Latino/a, Other). A dichotomous variable was created to differentiate between individuals born in the United States and individuals born elsewhere.

Sexual orientation (asexual, bisexual, gay, lesbian, pansexual, queer, questioning, same gender loving, straight/heterosexual, other) was collapsed to a three-category variable (straight, bisexual, other). Finally, two ordinal-level variables, mother's education and father's education (less than high school completed, high school diploma or equivalent, some college, vocational or trade school, bachelor's degree, master's degree, professional degree, doctorate, do not know, not applicable), were combined to create a “first generation” college students variable. Respondents who did not have a parent with a college degree were considered “first generation”; respondents with at least one parent with a college degree or higher were not considered “first generation.”

#### Symptoms suggestive of endometriosis

Symptoms suggestive of endometriosis were assessed by using a 26-measure scale adapted from prior research on adolescent menstrual health research.^37^ The scale began by asking, “Over the past 12 months have you experienced any of the following symptoms in relation to your period?” and then listed symptoms, for example, “vomiting,” “lower back pain,” and “pain during or after sexual intercourse.” After each symptom, response items were “no or never,” “just before a period,” “at the time of period,” “any time of the month,” “all of the time,” and “sometimes.”

If participants answered “no or never” for a symptom, they were coded as not having that symptom. If they selected any other response, they were coded as having that symptom. A count variable (with a minimum of 0 and a maximum of 26) was constructed to assess the number of symptoms that each respondent reported.

#### Disruptions

##### Academic disruptions

Participants were asked whether in the past year they missed school, missed an exam, or changed their course schedule because of symptoms associated with their period. They were also asked whether they delayed enrolling in college, had ever withdrawn from college, or changed colleges because of symptoms associated with their period. Responses for each question included “yes,” “no,” and “don't know.” A dichotomous “any academic disruption” variable was created such that respondents answered “yes” to any of the academic disruption questions versus “no” to all.

##### Social disruptions

Participants were asked (in separate questions) whether the symptoms associated with their period negatively interfered with: social activities, relationship and activities with family, relationship and activities with friends, relationship and activities with romantic partner, sexual activity, sports and exercise, and extracurricular activities. Response items for each were “yes,” “no,” and “don't know.” A dichotomous “any social disruption” variable was created in the same manner as academic disruptions.

##### Work disruptions

Work disruptions were assessed by first asking how many hours per week the respondents work. Respondents who answered “0” were then asked when they were not working because of symptoms associated with their period. They could answer “yes,” “no,” or “don't know.” Respondents who reported working 1 or more hours per week were asked how many hours in the past year they had missed work because of symptoms associated with their period. A dichotomous “any work disruption” variable was created in the same manner.

##### Any disruption

An overall dichotomous “any disruption” variable was created such that respondents experienced any academic, social, or work disruptions versus no to all.

#### Anticipated stigma

Anticipated stigma was assessed by using a 12-item scale developed for use among people experiencing chronic illness.^19^ The scale was adapted for this study so that it examines respondent's perceived likelihood of experiencing negative behaviors from family and friends, employers, or health care workers as a result, specifically, of symptoms associated with their period (Chronbach's alpha = 0.89). The scale asks, “How likely is it that any friend or family member will treat you in the following ways because of symptoms associated with your period?” Example items include, “a friend or family member will be angry with you,” and “your employer will not promote you.” Responses ranged from 0 “very unlikely” to 4 “very likely.” A mean stigma score was calculated by adding up the total points for each respondent's answers and dividing by 12.

### Analysis

Descriptive statistics were used to examine frequencies and means (where appropriate) of all variables. Bivariate analysis using Pearson's chi square, *t*-tests, and one-way analysis of variance assessed the relationship between demographic variables and disruptions, demographic variables and anticipated stigma, and endometriosis symptoms and (separately) disruptions and anticipated stigma. Linear regressions were used to assess the relationship between disruptions and anticipated stigma. We ran four unadjusted regressions—each with one of the disruption variables as the predictor and anticipated stigma as the outcome.

We then ran adjusted regressions for each of the disruptions variables (with anticipated stigma as the outcome), controlling for demographic variables in the first four regressions, and also controlling for the number of endometriosis symptoms in second four regressions. All analyses were conducted in Stata v14.1.

## Results

### Overall sample and response rate

Our survey had a response rate of 35% and a final sample size of 468. The majority of our respondents, 65.6%, reported their race as White, whereas 16.9% reported their race as Black, 11.3% as Latina/Hispanic, and 6.2% reported another race. With a mean age of 20.6, more than three-fourths of our college-aged respondents were enrolled as full-time students at a university (76.9%) and 69% lived off campus. Almost half, 43.4%, were first-generation college students, and 93% were born in the United States.

Sixty-five percent of respondents reported their sexual orientation as straight/heterosexual, and 17.9% were in a relationship and cohabitating at the time of the survey; 45.9% reported being single. Regarding perceived health status, 20.2% of women rated their health as “excellent,” whereas 94% reported “fair/poor” health. Finally, our sample's mean EndoScore, representing the scores from the endometriosis symptoms scale, was 13.7 (standard deviation [SD] = 5.6).

### Disruptions and bivariate associations with demographics

Among our sample, 88% experienced any disruptions related to symptoms consistent with endometriosis in the past year (Table 1). Social disruptions were most frequently reported (82.7%), followed by academic disruptions (58.8%) and work disruptions (34.4%).

**Table 1.** Bivariate Associations Between Demographics, Disruptions, and Anticipated Stigma (***N* = 468)**

**Demographic variables**	**Sample** ***N* (%)^a^**	**Any disruption** ***n* (%)^b^**	***p***	**Academic disruption** ***n* (%)^b^**	***p***	**Social disruption** ***n* (%)^b^**	***p***	**Work disruption** ***n* (%)^b^**	***p***	**Anticipated stigma** **Mean (SD)^b^**	***p***
Total sample, *n* (%)	468 (100.0)	412 (88.0)		275 (58.8)		387 (82.7)		161 (34.4)		2.03 (1.85)	
Age (mean = X)	20.6	20.5	0.492	20.5	0.433	20.5	0.176	20.7	0.256	−0.03^c^	0.763
Race											
White	307 (65.6)	87.3	0.498	55.7	0.051	82.4	0.925	28.0	<0.001	1.96	0.074
Black	79 (16.9)	92.4		65.8		83.5		45.6		2.17	
Latino/a	53 (11.3)	88.7		71.7		84.9		62.3		2.21	
Other	29 (6.2)	82.8		48.3		79.3		20.7		2.04	
Country of origin											
U.S.	435 (93.0)	88.5	0.254	58.2	0.339	82.8	0.890	33.3	0.077	2.01	0.050
Other	33 (7.0)	81.8		66.7		81.8		48.5		2.30	
Sexual orientation											
Straight	305 (65.2)	85.9	0.065	54.8	0.055	81.3	0.109	30.2	0.003	1.87	<0.001
Bisexual	84 (17.9)	95.2		66.7		90.5		50.0		2.35	
Other	79 (16.9)	88.6		65.8		79.8		34.2		2.03	
First-generation college											
Yes	203 (43.4)	86.7	0.436	60.1	0.607	82.7	0.974	39.4	0.046	2.04	0.688
No	265 (56.6)	89.1		57.7		82.6		30.6		2.01	
Enrollment status											
Full-time	360 (76.9)	87.2	0.323	56.1	0.034	82.5	0.841	29.7	<0.001	1.98	0.037
Part-time	108 (23.1)	90.7		67.6		83.3		50.9		2.17	
Living situation											
Off campus	323 (69.0)	85.5	0.010	57.3	0.330	80.2	0.032	34.4	0.980	2.03	0.698
On campus	145 (31.0)	93.8		62.1		88.3		34.5		2.00	
Relationship status											
Single	215 (45.9)	87.4	0.082	55.8	0.387	80.9	0.162	36.3	0.044	2.02	0.035
Relationship, not cohabitating	169 (36.1)	91.7		59.8		87.0		27.8		1.94	
Relationship, cohabitating	84 (17.9)	82.1		63.3		78.6		42.9		2.23	
Health status											
Excellent	86 (20.2)	87.2	0.515	48.8	0.075	77.9	0.182	39.5	0.031	1.86	0.007
Very good	167 (39.3)	85.0		53.3		80.8		24.0		1.91	
Good	132 (31.1)	89.4		62.9		84.9		37.2		2.05	
Fair/Poor	40 (9.4)	92.5		67.5		92.5		30.0		2.34	
Endometriosis symptoms											
EndoScore, mean (SD)	13.7 (5.6)	14.4	<0.001	15.3	<0.001	14.5	<0.001	16.0	<0.001	0.70	<0.001

^a^ Column percentages.

^b^ Row percentages.

^c^ Beta coefficient.

SD, standard deviation.

#### Any disruptions

Women who lived on campus reported more “any disruption” in comparison to those who lived off campus (93.8% vs. 85.5%, respectively; *p* = 0.01). No other demographic variables were found to be significantly associated with any disruptions.

#### Social disruption

Social disruptions due to endometriosis symptoms significantly varied by living situation (*p* = 0.032), with 88.3% of on-campus students experiencing social disruptions compared with 80.2% of off-campus students. No other demographic variables emerged as significant.

#### Academic disruption

Academic disruptions varied by race (71.7% of Latinas and 65.8% of Black Women versus 55.7% of White women, *p* = 0.051), sexual orientation (66.7% of bisexual women experienced academic disruptions compared with 54.8% of straight women, *p* = 0.055), and enrollment status (67.6% of part-time students experienced academic disruptions compared with 56.1% of full-time students, *p* = 0.034).

#### Work disruption

Experiencing work disruptions differed significantly by many demographic variables, with 62.3% of Latinas and 45.6% of Black women reporting such disruptions versus 28% of White Women (*p* < 0.001). Sexual orientation was also associated with differences in experiences of work disruptions (*p* = 0.003); half of bisexual women experienced these disruptions, whereas 30.2% of straight women reported such disruptions. Thirty-nine percent of first-generation college students reported work disruptions compared with 30.6% of non-first-generation students (*p* = 0.046). Around half (50.9%) of part-time students experienced work disruptions, whereas around 30% of full-time students reported the same disruptions (*p* < 0.001). Additional findings can be seen in Table 1.

### Anticipated stigma and bivariate associations with demographics

The overall mean anticipated stigma score was 2.03 (SD = 1.85) (Table 1). Women born outside of the United States had higher anticipated stigma mean scores in comparison to their U.S.-born counterparts (2.30 vs. 2.01; *p* = 0.05). Regarding sexual orientation, bisexual women had a higher mean score compared with straight women (2.35 vs. 1.87; *p* < 0.001). Higher anticipated mean scores were also observed among part-time students versus full-time students (2.17 vs. 1.98; *p* = 0.037). Finally, both relationship status and health status were significantly associated with differences in mean anticipated stigma (*p* = 0.035 and 0.007, respectively).

Women in a cohabitating relationship had a mean anticipated stigma score of 2.23, whereas women in a non-cohabitating relationship had a score of 1.94. As for self-reported health, women who reported excellent health had a lower mean anticipated stigma score compared with women who reported fair/poor health (1.86 vs. 2.84); *p*-values were 0.035 and 0.007, respectively.

### Multivariable associations between disruptions and anticipated stigma

In unadjusted analyses, reporting any disruptions, social disruptions, academic disruptions, and work disruptions was significantly associated with higher mean anticipated stigma scores (*β* = 0.67, 95% confidence interval [CI]: 0.45–0.90; *β* = 0.50, 95% CI: 0.40–0.78; *β* = 0.58, 95% CI: 0.44–0.72; *β* = 0.63, 95% CI: 0.48–0.77; respectively) (Table 2). After accounting for all demographic variables, these associations remained significant for any, social, academic, and work disruptions (*β* = 0.63, 95% CI: 0.41–0.86; *β* = 0.54, 95% CI: 0.35–0.74; *β* = 0.49, 95% CI: 0.34–0.63; *β* = 0.55, 95% CI: 0.38–0.71; respectively).

**Table 2.** Unadjusted and Adjusted Linear Regression Examining Associations Between Disruptions and Anticipated Stigma (***N* = 468)**

	**Model 1 (disruptions only)**			**Model 2 (adjusted for demographic covariates^a^)**			**Model 3 (adjusted for demographic covariates and symptoms suggestive of endometriosis^b^)**		
**Anticipated stigma**									
	**Coeff**	**SE**	**95% CI**	**Coeff**	**SE**	**95% CI**	**Coeff**	**SE**	**95% CI**
Disruptions									
Any disruptions	0.67^*^	0.10	0.45–0.90	0.63^**^	0.11	0.41–0.86	0.37^**^	0.11	0.15–0.59
Any school disruptions	0.58^*^	0.05	0.44–0.72	0.49^**^	0.07	0.34–0.63	0.32^**^	0.07	0.17–0.46
Any social disruptions	0.50^*^	0.09	0.40–0.78	0.54^**^	0.10	0.35–0.74	0.32^**^	0.01	0.13–0.51
Any work disruptions	0.63^*^	0.09	0.48–0.77	0.55^**^	0.08	0.38–0.71	0.38^**^	0.01	0.23–0.54

^*^ *p* < 0.001, ^**^p < 0.0001.

^a^ Adjusted for age, race, first-generation student, U.S. born, relationship status, enrollment, sexual orientation, living off campus.

^b^ Adjusted for symptoms suggestive of endometriosis and demographics (age, race, first generation student, U.S. born, relationship status, enrollment, sexual orientation, living off campus).

CI, confidence interval; SE, standard error.

Lastly, once endometriosis symptoms were added to the models adjusted for demographics, all associations were attenuated, but remained significant (*β* = 0.37, 95% CI: 0.15–0.59; *β* = 0.32, 95% CI: 0.13–0.51; *β* = 0.32, 95% CI: 0.17–0.46; *β* = 0.38, 95% CI: 0.23–0.54; respectively).

## Discussion

This study with college-attending women documented high levels of life disruptions due to symptoms suggestive of endometriosis. These disruptions spanned multiple domains of life, including social, academic, and work. The current findings are broadly consistent with existing data on life disruptions due to endometriosis among adult women. Our findings extend such research into this relatively under-researched population and underscore disruptions due to symptoms suggestive of endometriosis as a critical health concern impacting university-attending populations.

Newer to the body of work are our findings pertaining to anticipated stigma. Overall, we found a mean anticipated stigma score of 2.03 in the current sample. This is broadly consistent with existing research on anticipated stigma among other populations with chronic illnesses (*e.g.*, patients with fibromyalgia, inflammatory bowel disease).^24,25^ Existing research showcases how anticipated stigma is associated with multiple negative health outcomes, including poor mental health and lower help-seeking.^19,27,28^

Our finding of comparable levels of anticipated stigma, thus, underscores the need for more research and programmatic attention to addressing stigma due to symptoms suggestive of endometriosis to mitigate potential negative health impacts in this population during this critical developmental phase. The current study also signals the importance of future research to integrate an intersectional framework. Participants who were immigrants, sexual and gender minority women, and Black and Latina reported higher levels of anticipated stigma (in comparison to U.S.-born, straight/heterosexual, and White participants, respectively). Anticipated stigma may be shaped by structures within society that systematically marginalize the aforementioned groups. Historically, endometriosis research is highly skewed toward White populations, as are current advocacy efforts.

Also new to existing research is our finding regarding the relationships between disruptions due to symptoms suggestive of endometriosis and anticipated stigma. In the current sample, women who reported disruptions due to symptoms suggestive of endometriosis also reported higher levels of anticipated stigma. This relationship was consistently observed across all domains of disruptions examined in the current study (any, social, academic, work).

Moreover, disruptions were related to higher levels of anticipated stigma, even after accounting for the effect of symptoms that may be suggestive of endometriosis. More research is needed to better understand the potential mechanisms that may underlie such observed associations. Certainly, as shown in existing research, symptoms (*e.g.*, pain) have harmful impacts on emotional health.^39,40^ However, more understanding is needed as to why the associations between disruptions and higher levels of anticipated stigma continued to persist above and beyond the effect of symptoms.

The current study does not allow us to examine what specific aspects of disruptions may be most relevant to higher levels of anticipated stigma. Consistent with research on the stigma theory and concealable stigma, it may be that disruptions remain a critical stressor due to fear of disappointing others and/or fear of illness/symptoms being discovered by others.^18^ Endometriosis symptoms (*e.g.*, pain, bowel dysfunction) can often be concealed by staying home and/or “pretending” that things are fine. Women report concealing such symptoms due to fear of being judged or dismissed by others.^21,29^

Mixed-methods research may help provide additional insight into our observations. Moreover, future research on the interplay of anticipated stigma with other forms of stigma (*e.g.*, internalized, enacted) may also be helpful with advancing understanding of underlying mechanisms and processes.

The current study must be interpreted within the context of important limitations. First, this study was conducted with an online sample and was not probability-based. Thus, findings cannot be generalized to broader populations of college students. Further, due to the manner in which data were collected, we are unable to determine whether those who declined participation differed systematically from those who agreed to participate.

It is also important to note that data on surgically confirmed diagnoses are not in this current study. Endometriosis can only be definitively diagnosed with surgical biopsy confirmation. Thus, disruptions reported herein may also reflect other types of gynecologic pathology. However, young women experience diagnostic delays that range from 7 to 11 years.^10^ Lastly, although anticipated stigma was found to have strong psychometric properties in the current sample in terms of internal consistency reliability, the scale has not been validated in this sample. It may also be possible that participants reported on anticipated stigma for reasons other than disruptions due to symptoms suggestive of endometriosis.

## Conclusion

These limitations notwithstanding, the current study sought to examine broader psychosocial experiences, such as disruptions due to symptoms and associated anticipated stigma—a topic that is under-researched, especially with young adult women. Hence, this study has important implications for future research and public health efforts. Endometriosis research is woefully underfunded, and the majority of federal and private funding is skewed toward clinical research and treatment outcomes, with far less attention to psychosocial health.

Similarly, the small number of public outreach efforts focus primarily on health education and raising awareness, without adequate attention to addressing stigma in broader society (*e.g.*, workplaces, higher education, peer groups). Research-based interventions that explicitly address stigma within broader society are needed. Lessons learned from other stigma reduction interventions in the HIV and mental health arenas may offer critical insight for addressing stigma pertaining to disruptions and endometriosis.^41,42^

Moreover, findings suggest that in addition to increased investment in surgical and pharmaceutical treatments, increased investments are also needed to assist young adult women with navigating disruptions. This may include adapting patient navigator models for other chronic illnesses (*e.g.*, breast cancer, HIV). Such approaches have been helpful for navigating disruptions and other challenges, and they may benefit patients with endometriosis. More research and intervention efforts focused on stigma, and endometriosis have the potential to benefit women and girls living with this condition.

## Abbreviations Used

CI: confidence interval
SE: standard error

## References

[1] Eskenazi B, Warner ML. Epidemiology of endometriosis. Obstet Gynecol Clin North Am 1997;24:235–258. 9163765 10.1016/s0889-8545(05)70302-8

[2] Cramer DW, Missmer SA. The epidemiology of endometriosis. Ann N Y Acad Sci 2002;955:11–22. 11949940 10.1111/j.1749-6632.2002.tb02761.x

[3] Rogers PAW, D'Hooghe TM, Fazleabas A, et al. Priorities for endometriosis research: Recommendations from an International Consensus Workshop. Reprod Sci 2009;16:335–346. 19196878 10.1177/1933719108330568 PMC3682634

[4] Culley L, Law C, Hudson N, et al. The social and psychological impact of endometriosis on women's lives: A critical narrative review. Hum Reprod Update 2013;19:625–639. 23884896 10.1093/humupd/dmt027

[5] De Nardi PD, Ferrrari S. Diagnosis. In: Deep Pelvic Endometriosis: A Multidisciplinary Approach. Milan, Italy: Springer-Verlag Italia. 2011; pp. 17–45.

[6] Lemaire GS. More than just menstrual cramps: Symptoms and uncertainty among women with endometriosis. J Obstet Gynecol Neonatal Nurs 2004;33:71–79. 10.1177/0884217503261085 14971555

[7] Pluchino N, Wenger J-M, Petignat P, et al. Sexual function in endometriosis patients and their partners: Effect of the disease and consequences of treatment. Hum Reprod Update 2016;22:762–774. 27591248 10.1093/humupd/dmw031

[8] Yantiss RK, Clement PB, Young RH. Endometriosis of the intestinal tract: A study of 44 cases of a disease that may cause diverse challenges in clinical and pathologic evaluation. Am J Surg Pathol 2001;25:445–454. 11257618 10.1097/00000478-200104000-00003

[9] Jones CE. The pain of endo existence: Toward a feminist disability studies reading of endometriosis. Hypatia 2016;31:554–571.

[10] Nnoaham KE, Hummelshoj L, Webster P, et al. Impact of endometriosis on quality of life and work productivity: A multicenter study across ten countries. Fertil Steril 2011;96:366–373.e8. 21718982 10.1016/j.fertnstert.2011.05.090 PMC3679489

[11] Petrelluzzi KFS, Garcia MC, Petta CA, Grassi-Kassisse DM, Spadari-Bratfisch RC. Salivary cortisol concentrations, stress and quality of life in women with endometriosis and chronic pelvic pain: Research report. Stress 2008;11:390–397. 18800310 10.1080/10253890701840610

[12] Gilmour JA, Huntington A, Wilson HV. The impact of endometriosis on work and social participation. Int J Nurs Pract 2008;14:443–448. 19126072 10.1111/j.1440-172X.2008.00718.x

[13] Fourquet J, Báez L, Figueroa M, Iriarte RI, Flores I. Quantification of the impact of endometriosis symptoms on health-related quality of life and work productivity. Fertil Steril 2011;96:107–112. 21621771 10.1016/j.fertnstert.2011.04.095 PMC3129383

[14] Gupta J, Cardoso LF, Harris CS, et al. How do adolescent girls and boys perceive symptoms suggestive of endometriosis among their peers? Findings from focus group discussions in New York City. BMJ Open 2018;8:e020657. 10.1136/bmjopen-2017-020657 PMC5988150 29866728

[15] Link BG, Phelan JC. Conceptualizing stigma. Annu Rev Sociol 2001;27:363–385.

[16] Phelan JC, Link BG, Dovidio JF. Stigma and prejudice: One animal or two? Soc Sci Med 2008;67:358–367. 18524444 10.1016/j.socscimed.2008.03.022 PMC4007574

[17] Kurzban R, Leary MR. Evolutionary origins of stigmatization: The functions of social exclusion. Psychol Bull 2001;127:187–208. 11316010 10.1037/0033-2909.127.2.187

[18] Pachankis JE. The psychological implications of concealing a stigma: A cognitive-affective-behavioral model. Psychol Bull 2007;133:328–345. 17338603 10.1037/0033-2909.133.2.328

[19] Earnshaw V, Quinn D, Kalichman S, Park C. Development and psychometric evaluation of the Chronic Illness Anticipated Stigma Scale. J Behav Med 2013;36:270–282. 22526525 10.1007/s10865-012-9422-4 PMC4370181

[20] Cox H, Henderson L, Andersen N, Cagliarini G, Ski C. Focus group study of endometriosis: Struggle, loss and the medical merry-go-round. Int J Nurs Pract 2003;9:2–9. 12588614 10.1046/j.1440-172x.2003.00396.x

[21] Seear K. The etiquette of endometriosis: Stigmatisation, menstrual concealment and the diagnostic delay. Soc Sci Med 2009;69:1220–1227. 19699572 10.1016/j.socscimed.2009.07.023

[22] Denny E. Women's experience of endometriosis. J Adv Nurs 2004;46:641–648. 15154905 10.1111/j.1365-2648.2004.03055.x

[23] Markovic M, Manderson L, Warren N. Endurance and contest: Women's narratives of endometriosis. Health (London) 2008;12:349–367. 18579632 10.1177/1363459308090053

[24] Earnshaw VA, Quinn DM, Park CL. Anticipated stigma and quality of life among people living with chronic illnesses. Chronic Illness 2012;8:79–88. 22080524 10.1177/1742395311429393 PMC3644808

[25] Earnshaw VA, Quinn DM. The impact of stigma in healthcare on people living with chronic illnesses. J Health Psychol 2012;17:157–168. 21799078 10.1177/1359105311414952 PMC8919040

[26] Turan B, Budhwani H, Fazeli PL, et al. How does stigma affect people living with HIV? The mediating roles of internalized and anticipated HIV stigma in the effects of perceived community stigma on health and psychosocial outcomes. AIDS Behav 2017;21:283–291. 27272742 10.1007/s10461-016-1451-5 PMC5143223

[27] Earnshaw VA, Smith LR, Chaudoir SR, Amico KR, Copenhaver MM. HIV Stigma mechanisms and well-being among PLWH: A test of the HIV stigma framework. AIDS Behav 2013;17:1785–1795. 23456594 10.1007/s10461-013-0437-9 PMC3664141

[28] Quinn DM, Williams MK, Quintana F, et al. Examining effects of anticipated stigma, centrality, salience, internalization, and outness on psychological distress for people with concealable stigmatized identities. PLoS One 2014;9:e96977. 24817189 10.1371/journal.pone.0096977 PMC4016201

[29] Matías-González Y, Sánchez-Galarza AN, Flores-Caldera I, Rivera-Segarra E. “Es que tú eres una changa”: Stigma experiences among Latina women living with endometriosis. J Psychosom Obstet Gynaecol 2021:42:67–74. 10.1080/0167482X.2020.1822807 PMC8893272 32964770

[30] Jones NL, Gilman SE, Cheng TL, Drury SS, Hill CV, Geronimus AT. Life course approaches to the causes of health disparities. Am J Public Health 2019;109(S1):S48–S55. 30699022 10.2105/AJPH.2018.304738 PMC6356123

[31] Gee GC, Hing A, Mohammed S, Tabor DC, Williams DR. Racism and the life course: Taking time seriously. Am J Public Health 2019;109(S1):S43–S47. 30699016 10.2105/AJPH.2018.304766 PMC6356137

[32] Arnett JJ. Emerging adulthood: What is it, and what is it good for? Child Dev Perspect 2007;1:68–73.

[33] Vasquez-Tokos J, Norton-Smith K. Talking back to controlling images: Latinos' changing responses to racism over the life course. Ethn Racial Stud 2017;40:912–930.

[34] Zimmer-Gembeck MJ, Skinner EA. Review: The development of coping across childhood and adolescence: An integrative review and critique of research. Int J Behav Dev 2011;35:1–17.

[35] As-Sanie S, Black R, Giudice LC, et al. Assessing research gaps and unmet needs in endometriosis. Am J Obstet Gynecol 2019;221:86–94. 30790565 10.1016/j.ajog.2019.02.033

[36] Moradi M, Parker M, Sneddon A, Lopez V, Ellwood D. Impact of endometriosis on women's lives: A qualitative study. BMC Womens Health 2014;14:123. 25280500 10.1186/1472-6874-14-123 PMC4287196

[37] Parker MA, Sneddon AE, Arbon P. The menstrual disorder of teenagers (MDOT) study: Determining typical menstrual patterns and menstrual disturbance in a large population-based study of Australian teenagers. BJOG 2010;117:185–192. 19874294 10.1111/j.1471-0528.2009.02407.x

[38] ACHA-NCHA_IIc_Web_Survey_2011_SAMPLE.pdf. Available at: https://www.acha.org/documents/ncha/ACHA-NCHA_IIc_Web_Survey_2011_SAMPLE.pdf Accessed June 22, 2021.

[39] Brasil DL, Montagna E, Trevisan CM, et al. Psychological stress levels in women with endometriosis: Systematic review and meta-analysis of observational studies. Minerva Med 2020;111:90–102. 31755674 10.23736/S0026-4806.19.06350-X

[40] Estes SJ, Huisingh CE, Chiuve SE, Petruski-Ivleva N, Missmer SA. Depression, anxiety, and self-directed violence in women with endometriosis: A retrospective matched-cohort study. Am J Epidemiol 2021;190:843–852. 33184648 10.1093/aje/kwaa249 PMC8247611

[41] Nyblade L, Mingkwan P, Stockton MA. Stigma reduction: An essential ingredient to ending AIDS by 2030. Lancet HIV 2021;8:e106–e113. 33539757 10.1016/S2352-3018(20)30309-X

[42] Eiroa-Orosa FJ, Lomascolo M, Tosas-Fernández A. Efficacy of an intervention to reduce stigma beliefs and attitudes among primary care and mental health professionals: Two cluster randomised-controlled trials. Int J Environ Res Public Health 2021;18:1214. 33572955 10.3390/ijerph18031214 PMC7908111

